# Caseload midwifery compared to standard or private obstetric care for first time mothers in a public teaching hospital in Australia: a cross sectional study of cost and birth outcomes

**DOI:** 10.1186/1471-2393-14-46

**Published:** 2014-01-24

**Authors:** Sally K Tracy, Alec Welsh, Bev Hall, Donna Hartz, Anne Lainchbury, Andrew Bisits, Jan White, Mark B Tracy

**Affiliations:** 1 Midwifery and Women’s Health Research Unit, Royal Hospital for Women, Barker Street, Randwick, New South Wales 2031, Australia; 2 Department of Maternal Fetal Medicine, University of New South Wales, Randwick, New South Wales 2031, Australia; 3 Royal Hospital for Women, Barker Street, Randwick, New South Wales 2031, Australia; 4 Centre for Newborn Care, Westmead Hospital, Cnr Hawkesbury & Darcy Roads, Westmead, New South Wales 2145, Australia; 5 University of Sydney, Sydney, New South Wales 2006, Australia

**Keywords:** Midwifery group practice, Cost of caseload, Private obstetrics

## Abstract

**Background:**

In many countries midwives act as the main providers of care for women throughout pregnancy, labour and birth. In our large public teaching hospital in Australia we restructured the way midwifery care is offered and introduced caseload midwifery for one third of women booked at the hospital. We then compared the costs and birth outcomes associated with caseload midwifery compared to the two existing models of care, standard hospital care and private obstetric care.

**Methods:**

We undertook a cross sectional study examining the risk profile, birth outcomes and cost of care for women booked into one of the three available models of care in a tertiary teaching hospital in Australia between July 1st 2009 December 31st 2010. To control for differences in population or case mix we described the outcomes for a cohort of low risk first time mothers known as the 'standard primipara'.

**Results:**

Amongst the 1,379 women defined as 'standard primipara' there were significant differences in birth outcome. These first time ‘low risk’ mothers who received caseload care were more likely to have a spontaneous onset of labour and an unassisted vaginal birth 58.5% in MGP compared to 48.2% for Standard hospital care and 30.8% with Private obstetric care (p < 0.001). They were also significantly less likely to have an elective caesarean section 1.6% with MGP versus 5.3% with Standard care and 17.2% with private obstetric care (p < 0.001). From the public hospital perspective, over one financial year the average cost of care for the standard primipara in MGP was $3903.78 per woman. This was $1375.45 less per woman than those receiving Private obstetric care and $1590.91 less than Standard hospital care per woman (p < 0.001). Similar differences in cost were found in favour of MGP for all women in the study who received caseload care.

**Conclusions:**

Cost reduction appears to be achieved through reorganising the way care is delivered in the public hospital system with the introduction of Midwifery Group Practice or caseload care. The study also highlights the unexplained clinical variation that exists between the three models of care in Australia.

## Background

Australia's national caesarean section rate of 30.8% in 2011 sits above the OECD average of 25.8% of births [1] and well outside the World Health Organisation (WHO) recommendation of 15% [2]. This rate is increasing in both the public and private sectors in Australia, but continues to show a significant degree of unexplained clinical variation [3] and be substantially higher in the private sector [4-6].

In addition to the potential long term morbidity following caesarean section [7-10], operative birth incurs a measurable increase in cost [11,12], and an unquantified burden on the health system through pressure on resources such as staff and operating theatres [13]. The apparent inevitability of a rising caesarean rate due to the broadening indications for a primary caesarean is driving worldwide interest to find ways to address the issue [14].

Many countries have responded to this perceived public health concern with policies designed to promote a lower rate of operative birth and increase the rate of normal vaginal birth. In the US, the *Healthy People 2020* reports a national objective to reduce caesarean births among low risk first time mothers at full-term by 10% to 23.9 percent over the next ten years [15]. Similar policies have been promoted in the UK [16,17]. In New South Wales, Australia the 'Towards Normal Birth' policy directive was launched with the explicit aim of increasing the vaginal birth rate and decreasing the caesarean section rate [18].

At our tertiary hospital in New South Wales we introduced caseload midwifery care with a view to altering the caesarean section rate.

The latest Cochrane systematic review of midwife led care [19] recommends providing midwife led models of care to women in view of their known effectiveness, with a caveat that women who have complex pregnancies proceed with caution. However a randomised controlled trial of caseload midwifery care recently published in the Lancet concluded that for women of all risk caseload midwifery care costs less with similar clinical outcomes [20]. That study argued that caseload midwifery appeared to alter some of the pathways that recurrently contribute to increased obstetric intervention, working on the assumption that women will labour more effectively, need to stay in hospital less time and feel a stronger sense of satisfaction and personal control if they have the opportunity to get to know their midwife at the beginning of pregnancy.

The current project was also set in an Australian context with a similar population to that recently described in the randomised controlled trial of caseload care published in The Lancet [20]. The current study differed from the trial in that women were able to choose to have caseload care or standard care rather than be randomised to either model. In addition we also included in this analysis a third group of women – those who choose to receive private obstetric care in the public hospital.

Caseload midwifery offers greater relationship continuity, by ensuring that childbearing women receive their ante, intra and postnatal care from one midwife or her/his practice partner [21]. The evaluation of One-to-One Midwifery practice in the UK showed that continuity of carer could improve women’s satisfaction with their care, give midwives greater job satisfaction, increase their autonomy, and reduce intervention rates [22,23].

In a clinical redesign of maternity services in 2008 [24], we implemented nine caseload midwifery group practices (MGP) with the aim of providing continuity of midwifery care to women regardless of their risk status at booking. Prior to this there were two main maternity models on offer at the hospital - standard public hospital care or private obstetric care in the public hospital. At the outset we planned to evaluate the introduction of caseload through comparing the new model with both the cost and clinical outcomes of all women who received maternity care at the hospital during the study time period.

Caseload care in our setting is characterised by midwives arranged in formally recognised group practices of four midwives who undertake the midwifery management and responsibility for the continuum of care through pregnancy, birth and postpartum for a specified caseload of women [23]. The 'named' midwife provides leadership in midwifery care within her scope of practice with arrangements between partner members of the midwifery group practice to provide cover for leave and time off. Consultation and referral occurs as necessary using the Australian Midwifery Consultation and Referral Guidelines [25]. Collaborative practice is encouraged between the MGP midwives and a nominated consultant obstetrician or with other medical colleagues. Unlike other midwifery models such as team or birth centre care there is no limitation to only care for women deemed to be 'low risk'. In addition to this the MGP midwives experience a level of flexibility through their annualised salary contracts which allows them to self-manage their work hours in response to individual woman's needs rather than the ward roster system.

In the S*tandard Care* model women receive their care from rostered midwives in discrete wards or clinics; public hospital obstetric care (staff and trainee obstetricians) and community based general medical practitioner care. In the *Private Obstetric* model women pay for the services of a private obstetrician and receive private antenatal care in the rooms of their obstetrician. During labour and birth management decisions are made by the private obstetrician. Women are cared for in the hospital ward or clinic setting by the rostered midwives and obstetric trainees who provide the routine or standard public hospital care. Midwifery care in all three models is funded through state based revenue via the acute health services budget funding for public hospitals.

Following the introduction of Midwifery Group Practice at our hospital we undertook a cross sectional study to examine both the cost of each model of care from the standpoint of the public health system and the maternal and infant outcomes. There has not been an economic analysis of the three models of care available in the Australian public hospital setting to date.

The population included all women who gave birth at the metropolitan teaching hospital between 1^st^ July 2009 and 31^st^ December 2010. In an effort to make a more meaningful comparison between the three different models of care we examined more closely a sub group of the population known as the 'standard primipara' [26-28] similar to that reported recently by Coulm et al. [29] in France. These women were considered low‒risk at the time of birth and were having their first baby. We examined the outcomes of each option for maternity care available to all women, and in particular to those described as the 'standard primipara'. The primary outcomes were the mode of birth defined as caesarean section, instrumental birth or unassisted vaginal birth; and the cost associated with providing this care per woman from the standpoint of the public hospital over one financial year 2009/10.

## Methods

The study population included all women who gave birth at a large metropolitan tertiary teaching hospital between the 1st July 2009 and the 31st December 2010. Data were entered into the *Obstetrix* hospital data system by the attending midwife and electronically collated and checked by the research midwives. For missing data and data that were not credible the notes were checked manually. Maternal factors available for analysis were age, parity, medical conditions (any or none reported), and obstetric complications (any or none reported) as well as mental health disorders. Labour onset was described as spontaneous, induced or none (where an elective caesarean was performed). Induction was achieved through the use of drugs or mechanical means (Foley catheter) plus amniotomy - but not amniotomy alone. Augmentation referred to the acceleration of labour after 4 cm dilatation. Data were collected for unassisted vaginal birth, instrumental birth including vacuum and forceps, caesarean section including elective (no labour leading to caesarean section) as well as in labour, epidural in the first stage of labour, episiotomy and perineal status following birth. Neonatal factors included multiple birth, gestational age, birth weight, Apgar scores at one and five minutes, resuscitation techniques and admission to special care baby unit or neonatal intensive care nursery. Women having a first baby (at 20 weeks or more of gestation) were analysed separately to those women with a previous birth because of the significant impact of the care and outcome of previous pregnancies on care in multiparous pregnancies. Gestational age was calculated from menstrual dates noted by the woman and usually confirmed in the first trimester through routine ultrasound dating.

The group of women identified as the "standard primipara" is defined in the international and Australian literature [26-28] as a 20-34 year old woman, giving birth for the first time, free of obstetric and specific medical complications, with a singleton presenting by the vertex. The infant is of normal weight (10-90^th^ centile for birthweight) and born between 37 and 41 completed weeks of pregnancy. Comparison of intervention rates in this group of women effectively controls for differences in population or case mix between groups [26,27,30].

The cost of care was calculated for all women and controlled for differences in the groups of women in each model by examining the cost for primiparous, multiparous and standard primipara separately. We itemized each hospital occasion of service over one financial year (2009/10). The costing branch at the hospital obtained expenditure data for actual and estimated direct and indirect costs from the various cost centres at the hospital. Direct costs were collected for clinical midwifery and obstetric time; operating rooms; pathology; imaging; wards; allied health; pharmacy; depreciation and direct 'on costs'. Indirect costs included: indirect clinical midwifery and obstetric time; operating rooms; pathology; imaging; wards; allied health; pharmacy and indirect depreciation. (These are standard mechanisms to attribute an average cost per ward per unit time adjusted for complexity, although some costs are directly attributed to the patient such as X‒rays). The costs presented in this paper are based on expenditure data received from the hospital financial system which provides detailed information about the number of services each woman receives during her hospital stay. The costs for all services used by each woman were then aggregated to determine the total patient cost for pregnancy, birth and postnatal stay (from booking visit to 6 weeks postnatally).

Perinatal mortality was reported for neonates where death occurred during the first 4 weeks of life in a live born infant regardless of gestation or birth weight per 1000 births [31]. Both early and late neonatal deaths were included in the analysis, because deaths due to events in labour may occur beyond the early neonatal period. The perinatal death rate is defined as fetal deaths (of at least 20 weeks gestation or at least 400 grams birth weight), and all neonatal deaths.

Local Human Research Ethics Committee (HREC) approval was obtained (SESIAHS‒NHN N10/220).

### Analysis

Associations between model of care and maternal, infant, and clinical factors were examined by contingency table analyses unless otherwise specified.

Congenital anomalies were removed from the denominator. When the numbers of events were small, we used Fisher’s exact test. Total costs for each woman were summarized as medians and means, with 95% confidence intervals for mean differences by group, analysed with ANOVA using STATA 12 [32] and examined separately for primiparous, multiparous and standard primipara.

## Results

We excluded 51 women who were not booked and who were transferred to the hospital under emergency conditions for special medical care from outlying rural districts and 3 women who planned a homebirth and were attended by privately practicing homebirth midwives. This left a sample population of 6,020 women who planned and gave birth at the hospital between 1st July 2009 and the 31st December 2010. There were small but significant demographic differences between women who received care within each of the three maternity models (Table 1). Private obstetricians cared for more women with multiple pregnancies and more women whose infants fell below the 10^th^ centile in birthweight as well as a higher percentage of women older than 35 years (Table 1). MGP midwives cared for women with a small but significantly lower risk profile who gave birth to infants more likely to be in the higher gestational age and birthweight centiles (Table 1). Women under Standard Hospital management were less frequently older than 35 years, more primiparous and with a higher risk profile than either of the other two groups (Table 1). After excluding the 182 women who had a multiple pregnancy 5838 women gave birth to a singleton infant (Table 2) of whom 1,950 (33.4%) women were cared for by MGP; 2655 (45.4%) women had Standard public hospital care and 1233 (21.1%) gave birth in the public hospital under Private Obstetric care (Table 2).

**Table 1:** **Maternal and infant characteristics of all women who gave birth at the teaching hospital, 1**^**st **^**July 2009-31**^**st **^**December 2010**

	**MGP**	**Standard hospital**	**Private obstetric**
	**N = 1,965**	**N = 2,751**	**N = 1,304**
	**(32.8)**	**(45.6)**	**(21.6)**
	**%**	**%**	**%**
**Maternal age (years**)			
*Average age*	*32.4*	*31.7*	*34.0*
<20	1.5	1.4	0.3
20-34	63.5	67.2	51.6
> = 35	35.0	31.4	48.1
**Parity**			
Primiparous	49.3	55.4	52.6
Multiparous	50.7	44.6	47.4
**Any risk at onset of labour**			
None identified	73.1	57.7	61.9
Risk Identified	26.9	42.3	38.1
**BMI#**			
*Average*	*22.73*	*23.31*	*23.55*
Missing data	9.5	7.7	74.7
**Plurality**			
Singleton	99.2	96.5	94.6
Multiple	0.8	3.5	5.4
**Gestational age (weeks)**			
<37	5.0	11.3	14.3
37-41	93.4	88.0	85.7
42-43	1.6	0.7	0
**Birth weight (g)**			
<2500	3.2	9.3	11.2
2500-4499	94.9	89.2	87.8
> = 4500	1.9	1.5	1
**Birth weight percentiles**			
0-9.9	4.7	11.7	14.3
10.0-24.9	11.7	16.0	17.6
25.0-75.0	51.8	49.3	48.7
75.1-90.0	17.4	14.3	12.5
90.1-100	14.4	8.7	6.9

Values are in percentages.

Unless specifically stated the distribution of these variables is significantly (p <0.001) different between models of care using x^2^ tests.

# Analysis of variance was used to test differences in means across three groups with a Bonferroni correction.

**Table 2:** Labour and birth outcomes for all women who had a singleton pregnancy

	**MGP**		**Standard hospital**		**Private obstetric**		
	**n = 1950**		**n = 2655**		**n = 1233**		
	**No.**	**%**	**No.**	**%**	**No.**	**%**	
**‡Labour**							
Spontaneous onset	977	50.1	813	30.6	305	24.7	p < 0.001
Induction	373	19.1	736	27.7	321	26.0	p < 0.001
Augmentation	485	24.9	614	23.1	204	16.6	p < 0.001
**†Analgesia & 1**^**st **^**stage**							
None	642	32.9	386	14.5	118	9.6	
Epidural 1st stage	542	27.8	913	34.4	464	37.6	
Narcotic	144	7.4	316	11.9	44	3.5	
Nitrous O2	427	21.9	481	18.1	158	12.8	
Other	58	3.0	32	1.2	16	1.3	p < 0.001
**Mode of birth**							
Unassisted vaginal	1331	68.3	1304	49.1	450	36.5	
Instrumental birth	283	14.5	475	17.9	203	16.5	
C/S with labour	224	11.5	401	15.1	179	14.5	
Elective C/S (No labour)	112	5.7	475	17.9	401	32.5	p < 0.001
**Perineal status (excl. elective CS)**							
Intact following vaginal birth	462	25.0	669	30.1	295	34.3	
Grazes	167	9.0	142	6.4	29	3.4	
Episiotomy only	229	12.4	413	18.6	216	25.1	
1^st^ &2^nd^ degree tear	918	49.6	895	40.2	301	35.0	
3rd &4^th^ degree tear	52	2.8	61	2.7	7	0.8	
Episiotomy & 3/4^th^ degree tear	23	1.2	44	2.0	12	1.4	p < 0.001
**Infant outcomes**							
**Apgar score at 5 min**							
7-10	1909	97.9	2565	96.6	1205	97.8	
<7	41	2.1	90	3.4	28	2.3	p = 0.02
**Admission NICU/SCN**							
No	1,785	91.6	2,254	84.9	1076	87.3	
Yes	165	8.4	401	15.1	157	12.7	p < 0.001
**Outcome #**							
Live/survived	1937	99.60	2608	98.27	1218	98.94	
Live/neonatal death	1	0.05	21	0.79	4	0.32	
Stillbirth	7	0.36	25	0.94	9	0.73	p = 0.001

‡Percentages may not add up to 100% if women had induction and augmentation.

†Percentages may not add up to 100% if women had no analgesia before CS.

Distribution of these factors significantly (p < 0.001) different between models of care using x^2^ tests unless otherwise specified.

#Fishers exact test.

Amongst women with a singleton pregnancy (Table 2), those in MGP were significantly more likely to have a spontaneous onset of labour, less analgesia and a higher rate of vaginal birth with a lower admission rate to the neonatal and special care baby units (Table 2). Women with a singleton infant cared for by Private Obstetricians were more likely to have an elective caesarean (32.5%) than MGP (5.7%) or Standard hospital care (17.9%) (p < 0.001), and had a higher rate of epidural in the first stage of labour (37.6 versus 27.8) (p < 0.001) and a higher rate of episiotomy (31.4%) than MGP (11.6%) or Standard care (21.3%)(p < 0.001) (Table 2).

During the time of the study there were 1,379 (22.9%) women whom we described as the standard primipara (Table 3). *Standard primiparae* under MGP were significantly more likely to have a spontaneous onset of labour, experience an unassisted vaginal birth, and a lower rate of elective caesarean (1.6%) compared to Standard care (5.3%) and Private obstetric care (17.2%) p < 0.001 (Table 3).

**Table 3:** **Birth outcomes for the *'standard primipara' *** **associated with MGP, standard and private obstetric care**

	**MGP**		**Standard hospital**		**Private obstetric**		**MGP**		**Standard hospital**		**Private obstetric**		
	**N = 482**		**N = 647**		**N = 250**		**N = 482**		**N = 647**		**N = 250**		
**Labour & birth characteristics**	**N**	**(%)**	**95% CI**		**N**	**(%)**	**95%CI**		**N**	**(%)**	**95%CI**		
Spontaneous onset	217	(45.0)	40.6	49.5	207	(32.0)	28.5	35.7	70	(28.0)	22.8	33.9	p < 0.001
Augmentation	171	(35.5)	31.3	39.9	228	(35.2)	31.7-	39.0	67	(26.8)	21.7	32.6	p = 0.04
Epidural 1st stage	195	(40.5)	36.2	44.9	291	(45.0)	41.2	48.8	136	(54.4)	48.2	60.5	p = 0.002
**Mode of birth**													
Unassisted vaginal	282	(58.5)	54.1	62.9	312	(48.2)	44.3	52.1	77	(30.8)	25.1	36.5	
Instrumental birth	118	(24.5)	20.6	28.3	175	(27.0)	23.6	30.5	86	(34.4)	28.5	40.3	
C/S with labour	74	(15.4)	12.1	18.6	126	(19.5)	16.4	22.5	44	(17.6)	12.9	22.3	
Elective C/S (no labour)	8	( 1.6)	0.5	2.8	34	( 5.3)	3.5	6.9	43	(17.2)	12.5	21.9	p < 0.001
**Episiotomy**	101	(21.0)	17.3	24.6	171	(26.4)	23.0	29.8	76	(30.4)	24.7	36.1	p = 0.01
**Admit to NICU/SCN**	38	(7.9)	5.4	10.3	63	(9.7)	7.4	12.0	19	(7.6)	4.3	10.8	p = 0.48
**Apgar < 7 at 5 mins**	9	( 1.9)	0.66	3.1	14	(2.2)	1.0	3.2	2	(0.8)	0	1.9	p = 0.38

Public hospital costs calculated for the 4,038 women who received care within the three groups over one financial year are shown in Table 4. The average cost per woman per year receiving MGP care was $3,904.64. This was $1935.00 (95% CI $1,625.1‒$2,245.40) less than the woman receiving Standard care, and $1,394.88 (95% CI $1,019.90 ‒ $1,769.80) less than the woman receiving Private obstetric care (Table 4) (p < 0.001). (Note: this analysis does not include other costs to the taxpayer outside the public hospital system such as Medicare funding which is incurred by women receiving Private obstetric care or general practitioner shared care who receive antenatal care outside of the public hospital system.) The actual costs from the hospital perspective are further categorised for the care of primiparous women, multiparous women and the standard primipara showing mean, median, range, interquartile range and mean difference in costs (Table 4).

**Table 4:** Cost per woman from the public hospital perspective for one financial year 2009/10 in Australian dollars

**Model**	**mean**	**median**	**IQR**	**Mean diff (95% CI)**
**All women**				
**N = 4,038**				
**MGP (1,369)**	$3,904.64	$3,041.71	$1252.65 - $5593.33	Reference
**SC (1,799)**	$5,839.86	$5,159.50	$2850.95-$7603.85	$1,935 ($1,625.1 - $2,245.40)*
**POC (870)**	$5,299.52	$4994.69	$2728.47-$6744.90	$1,394.88 ($1,019.90 - $1,769.80)*
**Primiparous women**				
**N = 2,111**				
**MGP (671)**	$4722.11	$4124.37	$1887.07-$6664.49	Reference
**SC (983)**	$6307.02	$5879.71	$3577.41-$8045.18	$1,584.91 ($1167.60 - $2,002.20)*
**POC (457)**	$5878.80	$5775.72	$3512.11-$7186.27	$1,156.69 ($651.30 - $1,662.10)*
**Multiparous women**				
**N = 1,927**				
**MGP (698)**	$3118.79	$2121.75	$760.68-$4097.74	Reference
**SC (816)**	$5277.08	$4080.72	$2364.13-$7031.86	$2,158.29 ($1,704.70 - $2,611.90)*
**POC (413)**	$4658.53	$3922.21	$2435.91-$6163.57	$1,539.73 ($993.60 - $2,085.90)*
***Standard Primipara***				
***N = 963***				
**MGP (349)**	$3903.78	$3410.02	$1513.88-$5647.94	Reference
**SC (425)**	$5494.69	$5429.44	$3371.54-$6936.25	$1,590.91 ($1177.39- $2,004.43)*
**POC (189)**	$5279.23	$5218.63	$3505.79-$6633.25	$1,375.45 ($858.46 - $1,892.44)*

*p < 0.001.

IQR = Inter quartile range.

MGP = Midwifery group Practice; SC = Standard Care; POC = Private Obstetric Care.

(Note total population N = 4,038).

The characteristics of each model of care are outlined in Table 5.

**Table 5:** Factors differentiating midwifery and obstetric care in each model

	**Midwifery group practice (MGP) caseload care**	**Standard or routine hospital practice**	**Private obstetric care in the public hospital**
*Antenatal Care*	Women receive care with MGP midwives in the hospital/at home or in the community	Women receive care from the hospital antenatal clinic midwives or in combination with a GP and the hospital clinic midwives.	Women pay a fee and receive care from a private obstetrician in the obstetrician's rooms.
*When risks are identified during pregnancy*	Women continue to receive caseload midwifery care with the MGP midwife in consultation with a specialist clinic or with the obstetrician assigned to work with the Midwifery Group Practice.	Women are recommended to attend the doctor's clinic or a specialised clinic.	Women continue care with the private obstetrician or may be referred to a specialised clinic.
*When labour begins*	Women contact their MGP midwife and decide with their midwife when to go to the labour ward or birth centre.	Women contact labour ward and are advised via telephone whether to come in to the labour ward.	Women contact labour ward and are advised via telephone to come in. Labour ward staff alert the private obstetrician to the admission.
*Labour care*	Women are cared for by their known MGP midwife or her back-up partner. Problems are attended to by the registrar or consultant on call for birthing services.	Women are cared for by the rostered labour ward midwives. Problems are attended to by the registrar or consultant on call for birthing services.	Women are cared for by the rostered labour ward midwives in consultation with the private obstetrician. Urgent problems are attended to by the registrar on call until the private obstetrician arrives.
*Postnata*l	Women are discharged at 4 hours postnatal or after a short stay in the postnatal ward and visited by the MGP midwives.	Women are discharged to the home visiting service after a short ward stay.	Women stay in the postnatal ward until the private obstetrician discharges them home.
*Conditions of employment*	MGP midwives are employed on an annual salary which allows continuity of care for a caseload of women. They work in cycles of 152 hours over four (4) weeks; and do not work in excess of twelve (12) consecutive hours in any twenty four (24) hour period.	Midwives are rostered on wards or clinics and paid according to the award and whether they are full time (38 hours per week) or part time. They are employed to provide a rostered service.	Women booked under a private obstetrician receive the same public hospital midwifery care as those receiving Standard Care.

## Discussion

This small single centre cross sectional study found that MGP care is associated with significantly higher rates of 'normal birth' and a seemingly more cost effective method of delivering maternity care. This study is the first to compare cost and outcomes in the public hospital system in Australia associated with the three dominant models of care in large metropolitan centres. Factors that contributed to a lower cost were the increased rate of vaginal birth with fewer epidurals in the first stage of labour; lower rates of elective caesarean section, induction of labour, episiotomy and shorter postnatal lengths of stay.

The study is limited by size and selection bias where those women who chose MGP care may have a stronger commitment to achieving a normal vaginal birth outcome. However, in Australia as in other industrialised countries, women are positioned as self‒governing and autonomous consumers able to 'choose' what they consider their best option of care [33]. The introduction of MGP in the public hospital system could be seen as a further enhancement to women's choice and one that has the potential to provide the best market value for money in terms of public hospital funding. The study found an association between MGP care and fewer caesarean sections amongst women without complex pregnancies and having a first baby. Although these associations cannot be considered causal, information such as this is important for first time mothers for whom a first caesarean section so clearly establishes the direction of future pregnancy outcome [34]. To achieve a sustainable level of flexibility MGP midwives work within group practices of four midwives employed under a state approved annualised salary package which includes a 29% loading that provides an on-call allowance. They are required to work a cycle of 152 hours over a four week time period and do not work in excess of twelve consecutive hours in any twenty‒four hour period (24). MGP midwives may arrange their on call for alternate nights and weekends; or other configurations that are mutually agreed within the group practice. An integral factor in this model is the strong collaborative relationship between the MGP midwives and a nominated consultant obstetrician. Referral to medical or other services occurs as necessary using the Australian National Midwifery Consultation and Referral Guidelines [25].

## Conclusion

The Australian public are generally unaware of the association with model of care and birth outcomes. The latest Cochrane systematic review found that women who received continued care throughout pregnancy and birth from a small group of midwives were less likely to give birth pre-term and required fewer interventions during labour and birth than when their care was shared between different obstetricians, GPs and midwives [19]. Frequently the increased intervention rate within the private sector in Australia has been apportioned to the ‘higher risk’ population that seeks this care. By comparing a standardised low risk population: the standard primipara, we have shown that this may not be the case and that a level of unexplained variation exists in the care of maternity patients. Furthermore the results of this study demonstrate how cost reduction can be achieved through a radical system change in the way midwifery services are provided. A hypothetical scenario of the closure of two MGPs (320 women per annum) would increase the average cost of care at our hospital by $619.267.20 per year ($95% CI 520,032.00 ‒ 718,580.00).

Childbirth is the single most important reason for hospitalisation and accounts for the highest number of occupied bed days [34]; however, the current structure of our maternity system makes it challenging to deliver value for money. Financing arrangements, combined with the traditional case mix approach to public hospital funding, direct maternity care in Australia towards an acute care setting that uses specialist care and limits the role of midwives [35]. Large cost differences among women receiving care for similar conditions reveal additional opportunities for cost reduction [2,16]. Midwifery group practice models could play a major role in the future reducing the public health burden by increasing normal outcomes and promoting more efficient use of funds.

## Competing interests

The authors declare that they have no competing interests.

## Authors’ contributions

ST was responsible for the conceptual design of the study, drafted the manuscript and gave final approval of the version to be published. DH, AW, AB, AL and JW participated in the design of the study; helped draft the manuscript and participated in the day to day management and coordination of the study. MT participated in the study design; helped draft the manuscript performed the statistical analysis. JW is the overall manager of the midwifery group practices and participated in the study. BH participated in the design of the study; helped draft the manuscript and undertook the cost data linkages. All authors read and approved the final manuscript.

## Pre-publication history

The pre-publication history for this paper can be accessed here:

http://www.biomedcentral.com/1471-2393/14/46/prepub

## References

[B1] OECD “Caesarean sections”, in health at a glance 2011:OECD indicators, OECD publishing 2011 http://www.oecd-ilibrary.org/docserver/download/8111101ec037.pdf?expires=1390684600&id=id&accname=guest&checksum=11F56D1A7C7504FAE4602FC8054856D4

[B2] World Health Organisation Care in normal birth: a practical guide 1996 Geneva: Division of Family Health, WHO

[B3] Lee YY Roberts CL Patterson JA Unexplained variation in hospital caesarean section rates Med J Aust 2013 348 353 doi:10.5694/mja13.10279(199) 23992192 10.5694/mja13.10279

[B4] Laws P Li Z Sullivan EA Australia's mothers and babies 2008 2011 Canberra: AIHW

[B5] Roberts CL Tracy S Peat B Rates for obstetric intervention among private and public patients in Australia: population based descriptive study BMJ 2000 321 7254 137 141 10.1136/bmj.321.7254.137 10894690 PMC27430

[B6] Dahlen HG Tracy S Tracy M Rates of obstetric intervention among low-risk women giving birth in private and public hospitals in NSW: a population-based descriptive study BMJ Open 2012 0 e001723 doi:10.1136/bmjopen-2012-001723.accessed 23/01/2014 10.1136/bmjopen-2012-001723 PMC3467614 22964120

[B7] Cardwell CR Stene LC Joner G Caesarean section is associated with an increased risk of childhood-onset type 1 diabetes mellitus: a meta-analysis of observational studies Diabetologia 2008 51 5 726 735 10.1007/s00125-008-0941-z 18292986

[B8] Hyde MJ Mostyn A Modi N The health implications of birth by caesarean section Biol Rev 2011 doi:10.1111/j.1469-185X.2011.00195.x accessed 23/01/2014 10.1111/j.1469-185X.2011.00195.x 21815988

[B9] MacKay DF Smith GCS Dobbie R Pell JP Gestational Age at delivery and special educational need: retrospective cohort study of 407,503 schoolchildren PLoS Med 2010 7 6 e1000289 doi:10.1371/journal.pmed.1000289 accessed 23/01/2014 10.1371/journal.pmed.1000289 20543995 PMC2882432

[B10] Schlinzig T Johansson S Gunnar A Epigenetic modulation at birth – altered DNA-methylation in white blood cells after Caesarean section Acta Paediatr 2009 98 1096 1099 10.1111/j.1651-2227.2009.01371.x 19638013

[B11] Tracy SK Tracy MB Costing the cascade: estimating the cost of increased obstetric intervention in childbirth using population data BJOG 2003 110 8 717 724 10.1111/j.1471-0528.2003.02045.x 12892682

[B12] Allen VM O'Connell CM Farrell SA Economic implications of method of delivery Am J Obstet Gynecol 2005 193 1 192 197 10.1016/j.ajog.2004.10.635 16021078

[B13] Commonwealth of Australia Improving maternity services in Australia: report of the maternity services review 2009 http://www.health.gov.au/internet/main/publishing.nsf/Content/maternityservicesreview-report. Accessed 23/01/2014

[B14] Declercq E Young R Cabral H Is a rising cesarean delivery rate inevitable? Trends in industrialized countries, 1987 to 2007 Birth 2011 38 2 99 104 10.1111/j.1523-536X.2010.00459.x 21599731

[B15] US Department of Health and Human Services Healthy people 2020 2010 Washington DC: US Department of Health and Human Services

[B16] Werkmeister G Jokinen M Mahmood T Making normal labour and birth a reality.developing a multidisciplinary consensus Midwifery 2008 24 256 259 10.1016/j.midw.2008.06.001 18672321

[B17] NHS Insititute for Innovation and Improvement UK Promoting normal birth 2011 UK: NHS Institute for Innovation and Improvement, UK

[B18] New South Wales Health Maternity - towards normal birth in NSW PD2010_045 2011 Sydney, Australia: NSW Health

[B19] Sandall J Soltani H Gates S Shennan A Devane D Midwife-led continuity models versus other models of care for childbearing women Cochrane Database Syst Rev 2013 8 Art. No.: CD004667. doi:10.1002/14651858.CD004667.pub3. Accessed 23/01/2014 10.1002/14651858.CD004667.pub3 23963739

[B20] Tracy SK Hartz DL Tracy MB Allen J Forti M Hall B White J Lainchbury A Stapleton H Beckmann M Bisits A Homer C Foureur M Welsh A Kildea S Caseload midwifery care versus standard maternity care for women of any risk: M@NGO, a randomised controlled trial Lancet 2013 382 1723 1732 http://dx.doi.org/10.1016/S0140-6736(13)61406-3. See Online/Comment http://www.thelancet.com/journals/lancet/article/PIIS0140-6736(13)61793-6/fulltext 10.1016/S0140-6736(13)61406-3 24050808

[B21] McCourt C Stevens S Sandall J Page LA, McCandlish R Working with women: developing continuity in practice The new midwifery 2006 2 London: Churchill Livingstone Elsevier 141 167

[B22] Page L McCourt C Beake S Clinical interventions and outcomes of one-to-one midwifery practice J Public Health Med 1999 21 3 243 248 10.1093/pubmed/21.3.243 10528949

[B23] Page L One-to-one midwifery: restoring the "with woman" relationship in midwifery J Midwifery Womens Health 2003 48 2 119 125 10.1016/S1526-9523(02)00425-7 12686944

[B24] Hartz DL White J Lainchbury KA Australian maternity reform through clinical redesign Aust Health Rev 2012 36 2 169 175 10.1071/AH11012 22624638

[B25] Australian College of Midwives National midwifery guidelines for referral and consultation 2010 midwives.rentsoft.biz/…/Consultation%20and%20Referral%20Guidelines. Accessed 23/01/2014

[B26] Australian Council on Healthcare Standards (ACHS) Australasian clinical indicator report 2004–2011 2012 13 Sydney NSW: ACHS 78 84 http://www.achs.org.au/media/40455/achs_clinical_indicators_report_web.pdf Accessed 23/01/2014

[B27] Cleary R Beard RW Chapple J The standard primipara as a basis for inter-unit comparisons of maternity care Br J Obstet Gynaecol 1996 103 3 223 229 10.1111/j.1471-0528.1996.tb09710.x 8630306

[B28] Paterson CM Chapple JC Beard RW Evaluating the quality of the maternity services--a discussion paper Br J Obstet Gynaecol 1991 98 11 1073 1078 10.1111/j.1471-0528.1991.tb15357.x 1760417

[B29] Coulm B Le Ray C Lelong N Obstetric interventions for low-risk pregnant women in france: do maternity unit characteristics make a difference Birth 2012 39 3 183 191 10.1111/j.1523-536X.2012.00547.x 23281900

[B30] Knight M Sullivan EA Variation in caesarean delivery rates BMJ 2010 341 c5255 10.1136/bmj.c5255 20926492

[B31] Australian Bureau of Statistics Perinatal deaths, Australia, 2009 2011 Canberra: Australian Bureau of Statistics

[B32] StataCorp StataCorp. 2011. Stata statistical software: release 12 2012 USA: College Station, TX: StataCorp LP

[B33] Bryant J Porter M Tracy SK Caesarean birth: consumption, safety, order, and good mothering Soc Sci Med 2007 65 6 1192 1201 10.1016/j.socscimed.2007.05.025 17590252

[B34] Stavrou EP Ford JB Shand AW Epidemiology and trends for Caesarean section births in New South Wales, Australia: a population-based study BMC Pregnancy Childbirth 2011 11 8 10.1186/1471-2393-11-8 21251270 PMC3037931

[B35] Senate Community Affairs Reference Committee Rocking the cradle: a report of childbirth procedures 1999 Canberra: Commonwealth of Australia

